# Supplementary material for: Peoples’ understanding, acceptance, and perceived challenges of vaccination against COVID-19: A cross-sectional study in Bangladesh
Source: PLoS One. 2021 Aug 20;16(8):e0256493. doi: 10.1371/journal.pone.0256493 (PMC8378750; doi:10.1371/journal.pone.0256493)
Supplement: S1 Table — (DOCX) [file pone.0256493.s001.docx]

**S1 Table. Questionnaire on Peoples’ understanding and acceptability of COVID-19 vaccines, and perceived challenges on successful vaccination in Bangladesh.**

| **Socio-Demographic Information** | |
| --- | --- |
| **Questions** | **Responses** |
| 1. What is your gender? | - Men - Women |
| 2. What is your age (in years)? | - 18-30 - 31-40 - 41-50 - 51-60 - More than 60 |
| 3. What is your highest level of education? | - Illiterate - Up to Primary level - Up to SSC level - Diploma - Up to HSC level - Up to University level |
| 4. Where do you live now? | - City/ Town area - Sur-urban/upazila area - Rural area |

| **Knowledge about COVID-19 Vaccine** | |  |
| --- | --- | --- |
| **Questions** | **Responses** | **Reference** |
| 1. Have you heard about the COVID-19 vaccine? | - **Yes** - No |  |
| 2. How do you know about COVID-19 vaccine? | - **Newspapers** - **Television news** - **Social Media** - Friends or Colleagues - Family members |  |
| 3. Have you heard that COVID-19 vaccine is available in some countries? | - **Yes** - No |  |
| 4. Do you believe that vaccination can control COVID-19? | - **Yes** - No - Not Sure | [12, 14] |
| 5. Do you have any idea how many doses require for proper vaccination? | - One Dose - **Two Doses** - Not sure | [11, 34] |
| 6. Do you think that COVID-19 vaccine would have some side effects? | - **Yes** - No - Not Sure | [33] |
| 7. Which type of side effect may arise in the body after vaccination? | - **Primary side effects (Fever, Headache, Vomiting, etc.**) - Serious side effects (life-threatening) - No Idea | [33, 35] |
| 8. Which age group should be prioritized in receiving corona vaccine? | - **Old People** - Adult People - Children - Adolescent - All - Not Sure |  |
| 9. Which group should be prioritized in receiving vaccine? | - **Health Care Workers** - **People suffering from prolonged diseases (Diabetes, Cancer, Heart disease etc.)** - Non-COVID but Hospitalized Patients - Bureaucrats - Politicians - **Security Personals** - Teachers - Students - Not Sure |  |

| **Acceptability COVID-19 Vaccine** | |
| --- | --- |
| **Questions** | **Responses** |
| 1. Do you like to take COVID-19 vaccine? | - **Yes** - No |
| 2. Why do you like to take the COVID-19 vaccine? | - **I will be protected from COVID-19** - Government would suggest to take - Helps to reduce COVID-19 related fears - **Helps to reduce/control COVID-19 transmission** |
| 3. Which vaccine would you prefer? | - **Pfizer/BioNTech, USA** - Moderna, USA - **AstraZeneca, UK** - Sinovac, China - Sinopharm, China - Sputnik, Russia - **Covishield/ Serum Institute, India** - Covaxin/ Bharat Biotech, India - No Idea clarity |
| 4. If Bangladesh produces the COVID-19 vaccine, would you take it? | - **Yes** - No |
| 5. Why don’t you like to take the COVID-19 vaccine? | - **Religious issue** - **Possible Side effects** - **This will give temporary protection** - Might be expensive - Not necessary, I am fine - Will be protected from COVID-19 naturally |

| **COVID-19 Vaccine Management** | |
| --- | --- |
| **Questions** | **Responses** |
| 1. Do you think Bangladeshi authority would manage proper distribution of vaccine? | - **Yes** - No - Not Sure |
| 2. In your opinion, how vaccination should be implemented? | - Through NGOs - **Through Government Hospitals** - Through Private Clinics - Through Security Forces (e.g. Army, Navy - No Idea |
| 3. What would be the main challenges for COVID-19 vaccine management? | - **Motivating people to receive vaccine** - **Storage and Transport at Low Temperature** - Cost - Participant selection - Ensuring Vaccination Safety and Equipment - Coordination between Ministries and Field Level - No Idea |

Note: Bold responses were preferred answers
