# Supplementary material for: Peoples’ understanding, acceptance, and perceived challenges of vaccination against COVID-19: A cross-sectional study in Bangladesh
Source: PLoS One. 2021 Aug 20;16(8):e0256493. doi: 10.1371/journal.pone.0256493 (PMC8378750; doi:10.1371/journal.pone.0256493)
Supplement: S2 Table — (DOCX) [file pone.0256493.s002.docx]

**S2 Table. Category of logistic regression**

**Knowledge questions**

| Do you know/hear about COVID-19 vaccine? | Binary |
| --- | --- |
| **Yes** | **Yes** |
| No | No |
| Do you know/believe that vaccination can control COVID-19? |  |
| **Yes** | **Yes** |
| No | No |
| Not sure |  |
| Do you know how many doses require for proper vaccination? |  |
| One Dose | Others |
| **Two Doses** | **Yes (Right)** |
| Not sure | Others |
| Do you think that COVID-19 vaccines would have some side effects? |  |
| **Yes** | **Yes** |
| No | No |
| Not sure |  |
| Which type of side effects may arise in the body after vaccination? |  |
| Primary side effects (fever, headache, vomiting, etc.) | **Yes (Side effect)** |
| Serious side effects (life threatening) |  |
| No idea | No Idea |

**Vaccine acceptance questions**

| **Variable** |  | |
| --- | --- | --- |
| Do you like to take COVID-19 Vaccine | **Yes** | No |
| Reason (Protected from COVID) | **Yes** | No |
| Reason (Take and control transmission) | **Yes** | No |
| Bangladesh produces the COVID-19 vaccine, would you take it | **Yes** | No |
| Possible side effects and temporary protection | **Yes** | No |
| Not necessary, I am fine and protected naturally | **Yes** | No |
