# Supplementary material for: Peoples’ understanding, acceptance, and perceived challenges of vaccination against COVID-19: A cross-sectional study in Bangladesh
Source: PLoS One. 2021 Aug 20;16(8):e0256493. doi: 10.1371/journal.pone.0256493 (PMC8378750; doi:10.1371/journal.pone.0256493)
Supplement: S3 Table — (DOCX) [file pone.0256493.s003.docx]

**S3 Table. Omnibus Tests of Model Coefficients.**

Goodness of fit for different Logistic regression on Respondents’

knowledge of COVID-19 vaccination

| Heard about the Covid-19 Vaccine | | | | |
| --- | --- | --- | --- | --- |
|  |  | Chi-square | df | Sig. |
| Step 1 | *Step* | *457.702* | *16* | *0.000* |
|  | Block | 457.702 | 16 | 0.000 |
|  | Model | 457.702 | 16 | 0.000 |
| Believe that vaccine control COVID-19 | | | | |
|  |  | Chi-square | df | Sig. |
| Step 1 | *Step* | *133.781* | *16* | *0.000* |
|  | Block | 133.781 | 16 | 0.000 |
|  | Model | 133.781 | 16 | 0.000 |
| Dose | | | | |
|  |  | Chi-square | df | Sig. |
| Step 1 | *Step* | *835.720* | *16* | *0.000* |
|  | Block | 835.720 | 16 | 0.000 |
|  | Model | 835.720 | 16 | 0.000 |
| Side Effects | | | | |
|  |  | Chi-square | df | Sig. |
| Step 1 | *Step* | *582.842* | *16* | *0.000* |
|  | Block | 582.842 | 16 | 0.000 |
|  | Model | 582.842 | 16 | 0.000 |
| Type of Side Effect | | | | |
|  |  | Chi-square | df | Sig. |
| Step 1 | *Step* | *368.515* | *16* | *0.000* |
|  | Block | 368.515 | 16 | 0.000 |
|  | Model | 368.515 | 16 | 0.000 |

Goodness of fit for different Logistic regression on Respondents’

opinion of acceptance of COVID-19 vaccine

| Like to take COVID-19 Vaccine | | | | |
| --- | --- | --- | --- | --- |
|  |  | Chi-square | df | Sig. |
| Step 1 | *Step* | *745.730* | *20* | *0.000* |
|  | Block | 745.730 | 20 | 0.000 |
|  | Model | 745.730 | 20 | 0.000 |
| Reason (Protected from COVID) | | | | |
|  |  | Chi-square | df | Sig. |
| Step 1 | *Step* | *88.687* | *16* | *0.000* |
|  | Block | 88.687 | 16 | 0.000 |
|  | Model | 88.687 | 16 | 0.000 |
| Reason (Take and control transmission) | | | | |
|  |  | Chi-square | df | Sig. |
| Step 1 | *Step* | *269.740* | *16* | *0.000* |
|  | Block | 269.740 | 16 | 0.000 |
|  | Model | 269.740 | 16 | 0.000 |
| Bangladesh produces the COVID-19 vaccine, would you take it | | | | |
|  |  | Chi-square | df | Sig. |
| Step 1 | *Step* | *84.396* | *16* | *0.000* |
|  | Block | 84.396 | 16 | 0.000 |
|  | Model | 84.396 | 16 | 0.000 |
| Possible side effects and temporary protection | | | | |
|  |  | Chi-square | df | Sig. |
| Step 1 | *Step* | *135.549* | *16* | *0.000* |
|  | Block | 135.549 | 16 | 0.000 |
