# Supplementary material for: Peoples’ understanding, acceptance, and perceived challenges of vaccination against COVID-19: A cross-sectional study in Bangladesh
Source: PLoS One. 2021 Aug 20;16(8):e0256493. doi: 10.1371/journal.pone.0256493 (PMC8378750; doi:10.1371/journal.pone.0256493)
Supplement: Attachment — Submitted filename: Response to Reviewers.doc [file pone.0256493.s004.doc]

PONE-D-21-12921
Peoples’ understanding, acceptance, and perceived challenges of vaccination against COVID-19: Across-sectional study in Bangladesh

**Response to Reviewers’/Editor’s Comments**

Thanks, Academic Editor for inviting us to revise this paper. We have looked at editorial and reviewers’ comments. These are very constructive and useful to improve further our paper. We thank both reviewers for their time to read and comment on our paper. We have addressed all comments in this revision. Below please see our point-to-point responses:

**Response to Editor’s Comments**

Comment: Thank you for submitting your manuscript to PLOS ONE. After careful consideration, we feel that it has merit but does not fully meet PLOS ONE’s publication criteria as it currently stands. Therefore, we invite you to submit a revised version of the manuscript that addresses the points raised during the review process.

Response: Thanks for inviting us to submit the revision. We have addressed all points raised by the reviewers.

Comment: A rebuttal letter that responds to each point raised by the academic editor and reviewer(s). You should upload this letter as a separate file labeled 'Response to Reviewers'.

Response: We have addressed all points raised by the academic editor and reviewers in this rebuttal letter labelled as “Response to Reviewers”.

Comment: A marked-up copy of your manuscript that highlights changes made to the original version. You should upload this as a separate file labeled 'Revised Manuscript with Track Changes'.

Response: We have followed this instruction.

Comment: An unmarked version of your revised paper without tracked changes. You should upload this as a separate file labeled 'Manuscript'.

Response: We have followed this instruction.

Comment: If you would like to make changes to your financial disclosure, please include your updated statement in your cover letter. Guidelines for resubmitting your figure files are available below the reviewer comments at the end of this letter.

Response: We have not change financial statement.
Comment: If applicable, we recommend that you deposit your laboratory protocols in [protocols.io](http://protocols.io/) to enhance the reproducibility of your results. Protocols.io assigns your protocol its own identifier (DOI) so that it can be cited independently in the future.

Response: This is not applicable for our paper.

 Comment: Please ensure that your manuscript meets PLOS ONE's style requirements, including those for file naming.

Response: We have followed PLOS ONE’s style in formatting the paper and file naming as well.

Comment: In the Methods section please provide additional information regarding:
1) the participant eligibility criteria

Response: The inclusion criteria were being a Bangladeshi resident aged 18 years or over and living in Bangladesh at the time of the survey.

2) A justification for the sample size used in your study, including any relevant power calculations (if applicable).

Response: A total of 4175 responses (1975 online and 2200 face-to-face) were	obtained, which was satisfactory at the 95% confidence level with a ±5% margin of	error [30]. Israel [31] suggested a sample size of 400 at a 95% confidence level with a	±5% precision level. For α = 0.05 and a hypothesized proportion of 0.5, the power of	a sample size of 4175 is 1.0 (calculated using theSigmaXL statistical tool).

3) Steps taken to validate the questionnaire.

Response: In order to validate the questionnaire, six relevant experts were asked to	assess its relevance for this study. The relevance of a questionnaire has been widely	used to measure content validity [24 - 27]. All experts reported that the items	(questions) and responses were relevant to achieve the objectives of this study.

Comment: Furthermore when reporting the results of qualitative research, we suggest consulting the COREQ guidelines: <http://intqhc.oxfordjournals.org/content/19/6/349>. In this case, please consider including more information on the number of interviewers, their training and characteristics; and please provide the interview guide used. Please provide additional information regarding the type of informed consent taken for the qualitative study (i.e. written, verbal). Finally, please provide additional information regarding how the qualitative data were analysed.

Response: Following COREQ guidelines [29], seven in-depth telephone interviews were conducted with officials of health administration of Bangladesh (civil surgeons, divisional health directors, medical college hospital directors, and public health experts) to collect qualitative data. One of the authors, who had social medicine expertise, conducted the interviews. Interviews were guided by a check-list consisting of open-ended questions related to the **challenges, acceptance, confidence in vaccine management,** and the government’s future plans regarding vaccine safety. Interviewees were selected deliberately based on their engagement in the public health sector. Before each interview, informed verbal consent to participate in this study was obtained.

Comment: We suggest you thoroughly copyedit your manuscript for language usage, spelling, and grammar. If you do not know anyone who can help you do this, you may wish to consider employing a professional scientific editing service.  

Whilst you may use any professional scientific editing service of your choice, PLOS has partnered with both American Journal Experts (AJE) and Editage to provide discounted services to PLOS authors. Both organizations have experience helping authors meet PLOS guidelines and can provide language editing, translation, manuscript formatting, and figure formatting to ensure your manuscript meets our submission guidelines. To take advantage of our partnership with AJE, visit the AJE website (http://learn.aje.com/plos/) for a 15% discount off AJE services. To take advantage of our partnership with Editage, visit the Editage website ([www.editage.com](http://www.editage.com/)) and enter referral code PLOSEDIT for a 15% discount off Editage services.  If the PLOS editorial team finds any language issues in text that either AJE or Editage has edited, the service provider will re-edit the text for free.

 Response: An editor from EDITAGE edited this revised paper. A letter from EDITAGE is uploaded.

Comment: Upon resubmission, please provide the following:

- The name of the colleague or the details of the professional service that edited your manuscript

Response: A letter is uploaded.

- A copy of your manuscript showing your changes by either highlighting them or using track changes (uploaded as a *supporting information* file)

Response: We have done accordingly

- A clean copy of the edited manuscript (uploaded as the new *manuscript* file)

Response: We have done accordingly

**Response to Reviewers' Comments**

Reviewer's Responses to Questions

**Comments to the Author**

1. Is the manuscript technically sound, and do the data support the conclusions?

The manuscript must describe a technically sound piece of scientific research with data that supports the conclusions. Experiments must have been conducted rigorously, with appropriate controls, replication, and sample sizes. The conclusions must be drawn appropriately based on the data presented.

Reviewer #1: Yes

Reviewer #2: Yes

2. Has the statistical analysis been performed appropriately and rigorously?

Reviewer #1: Yes

Reviewer #2: Yes

3. Have the authors made all data underlying the findings in their manuscript fully available?

The [PLOS Data policy](http://www.plosone.org/static/policies.action#sharing) requires authors to make all data underlying the findings described in their manuscript fully available without restriction, with rare exception (please refer to the Data Availability Statement in the manuscript PDF file). The data should be provided as part of the manuscript or its supporting information, or deposited to a public repository. For example, in addition to summary statistics, the data points behind means, medians and variance measures should be available. If there are restrictions on publicly sharing data—e.g. participant privacy or use of data from a third party—those must be specified.

Reviewer #1: Yes

Reviewer #2: Yes

4. Is the manuscript presented in an intelligible fashion and written in standard English?

PLOS ONE does not copyedit accepted manuscripts, so the language in submitted articles must be clear, correct, and unambiguous. Any typographical or grammatical errors should be corrected at revision, so please note any specific errors here.

Reviewer #1: No

Reviewer #2: Yes

Response: Thanks for your positive feedback. We have asked a professional service provider to check English.

**Reviewer #1**

Comments on Abstract

- Back ground
- The research gap was not indicated

Response: We have added a sentence to justify this:

In order to eliminate COVID-19, many countries provided vaccinations. However, success depends on peoples’ knowledge levels and rates of acceptance. But, previous research on this topic is currently lacking in Bangladesh. This cross-sectional study aimed at to investigate Bangladeshi peoples’ knowledge, acceptance, and perception of challenges regarding COVID-19 vaccines.

Comments on Results

Comment: The proportions of outcome should be written along with 95% CI.

Response: We have checked and corrected all.

Comments: Why did sample size was wide? CI 11.40- 55 78.90; p=0.01) and their correct doses (OR 27.34; 95% CI 15.25-49.00; p=0.01)???

Response: This was due to (university) level of education which had important	influence on vaccines related knowledge.

Comment: Female (OR 1.16; 95% CI 0.96-1.40) was not statistically significant?

Response: Yes

Comments on Main body

Comment: Sample size determination was not clear and How do ensure data quality?

Response: We have described these as below:

**Questionnaire Development and Validation**

As questionnaires appropriate for this study, particularly the Bangladesh context, were not available, an original questionnaire was developed. A questionnaire was drafted based on the authors’ prior research experience and mass media information, which was reviewed by two experts and underwent a preliminary evaluation (pre-test) with 70 respondents. Based on experts’ comments and pre-test feedback, some questions were eliminated or rephrased for clarity. The final structured questionnaire consisted of 21 multiple choice questions in four categories: (1) socio-demographic information, includingfour questions, (2) knowledge of **COVID-19** vaccines,including nine questions, (3) acceptance of **COVID-19** vaccines,including five questions, and (4) **COVID-19** vaccine management,including three questions (S1 Table). The questionnaire was prepared in English and translated into *Bengali*, the national language of Bangladesh. In order to validate the questionnaire, six relevant experts were asked to assess its relevance for this study. The relevance of a questionnaire has been widely used to measure content validity [24 - 27]. All experts reported that the items (questions) and responses were relevant to achieve the objectives of this study.

**Sample Size and Power Calculation**

A total of 4175 responses (1975 online and 2200 face-to-face) were obtained, which was satisfactory at the 95% confidence level with a ±5% margin of error [30]. Israel [31] suggested a sample size of 400 at a 95% confidence level with a ±5% precision level. For α = 0.05 and a hypothesized proportion of 0.5, the power of a sample size of 4175 is 1.0 (calculated using theSigmaXL statistical tool).

Comment: Pre-test was not made

Response: Actually, we did pre-test and was not clearly described in this paper. We have now explained this.

Comment: How did you assess outcomes?

Response: We have assessed outcomes by odds ratio of logistic regressions.

Comment: The authors didn’t check internal consistency of tools.

Response: Yes, we did not check internal consistency. However, we validated questionnaire through experts’ comments on relevance of this questionnaire.

Comment: Confounding variables was not controlled for possible confounders and identified.

Response:Yes. Confounding variables was not controlled. We have taken only reliable independent variables in our study.

Comment: Multicollinearity was not checked.

Response: Multicollinearity test is generally performed for linear regression, and for logistic regression this is not required. So, we did not check this.

Comment: Which test is used to check model fitness?

Response: To check model fitness, the omnibus chi-square test was used and all models were found to be highly significant (S3 Table). The significance value of less than 0.05 indicated that the current model outperformed the null model. In all models, significant chi-square values indicated that the fitted model was better than the null model.

What is the mean(SD) of study subjects?

Response: Since our variables were categorical, we did not estimate mean (SD).

**Reviewer #2**

This very good article, well written this my commentts

Response: Thanks for your positive notes.

Comment: Abstract is long and need to be shorten

Response: We have tried shortened.

Comment: Methods: first please add paragraph about sample size calculation and techniques,

Response: We have described as below:

**Sample Size and Power Calculation**

A total of 4175 responses (1975 online and 2200 face-to-face) were obtained, which was satisfactory at the 95% confidence level with a ±5% margin of error [30]. Israel [31] suggested a sample size of 400 at a 95% confidence level with a ±5% precision level. For α = 0.05 and a hypothesized proportion of 0.5, the power of a sample size of 4175 is 1.0 (calculated using theSigmaXL statistical tool).

Comment: Regrading study tool please describe steps of construction and validation of questionnaire

Response: We have clarified these as below:

**Questionnaire Development and Validation**

As questionnaires appropriate for this study, particularly the Bangladesh context, were not available, an original questionnaire was developed. A questionnaire was drafted based on the authors’ prior research experience and mass media information, which was reviewed by two experts and underwent a preliminary evaluation (pre-test) with 70 respondents. Based on experts’ comments and pre-test feedback, some questions were eliminated or rephrased for clarity. The final structured questionnaire consisted of 21 multiple choice questions in four categories: (1) socio-demographic information, includingfour questions, (2) knowledge of **COVID-19** vaccines,including nine questions, (3) acceptance of **COVID-19** vaccines,including five questions, and (4) **COVID-19** vaccine management,including three questions (S1 Table). The questionnaire was prepared in English and translated into *Bengali*, the national language of Bangladesh. In order to validate the questionnaire, six relevant experts were asked to assess its relevance for this study. The relevance of a questionnaire has been widely used to measure content validity [24 - 27]. All experts reported that the items (questions) and responses were relevant to achieve the objectives of this study.

Comment: Regarding analysis, please mention the country of origin and manufactures of SPSS used

Response:SPSS for Windows, Version 16.0. Chicago, SPSS Inc.

Comment: Please remark correct answer or favorable responses for acceptable and perception questions

Response: We have done (Supplementary Table 1).
