# Supplementary material for: Peoples’ understanding, acceptance, and perceived challenges of vaccination against COVID-19: A cross-sectional study in Bangladesh
Source: PLoS One. 2021 Aug 20;16(8):e0256493. doi: 10.1371/journal.pone.0256493 (PMC8378750; doi:10.1371/journal.pone.0256493)
Supplement: Attachment — Submitted filename: Response to Reviewers.docx [file pone.0256493.s005.docx]

Peoples’ understanding, acceptance, and perceived challenges of vaccination against COVID-19: A cross-sectional study in Bangladesh

Thanks for inviting us for a minor revision of our revised paper. All comments are very much constructive and useful to improve our manuscript further. We have tried to address all comments. Please see below our responses:

**Academic Editor comments**:

1. Please include the following items when submitting your revised manuscript:

- A rebuttal letter that responds to each point raised by the academic editor and reviewer(s). You should upload this letter as a separate file labeled 'Response to Reviewers'.
- A marked-up copy of your manuscript that highlights changes made to the original version. You should upload this as a separate file labeled 'Revised Manuscript with Track Changes'.
- An unmarked version of your revised paper without tracked changes. You should upload this as a separate file labeled 'Manuscript'.

Response: We have done accordingly.

Journal Requirements:

2. Please review your reference list to ensure that it is complete and correct. If you have cited papers that have been retracted, please include the rationale for doing so in the manuscript text, or remove these references and replace them with relevant current references. Any changes to the reference list should be mentioned in the rebuttal letter that accompanies your revised manuscript. If you need to cite a retracted article, indicate the article’s retracted status in the References list and also include a citation and full reference for the retraction notice.

Response: We have checked all references carefully.

**Reviewers' comments**:

Reviewer #1: 

1. Background: State rephrase the objective of study

Response: Therefore, the objectives of this study were to explore the understanding and acceptance of COVID-19 vaccines, and perceptions regarding immunization challenges among people in Bangladesh.

2. Methods: indicate outcome measure

Response: We have added following description in methods:

This study examined two major outcome measures. First one was knowledge of COVID-19 vaccination of respondents (five dependent variables namely heard about the COVID-19 vaccine, believe that vaccine control COVID-19, dose, side effects, and type of side effects) using gender, age, resident and education as explanatory variables among the respondents. Second outcome measure was opinion of acceptance of COVID-19 vaccine of respondents (five dependent variables namely like to take COVID-19 vaccine; reason-protected from COVID-19; reason-take and control transmission; Bangladesh produces the COVID-19 vaccine, would you take it; and possible side effects and temporary protection) using gender, age, resident, education, believe that vaccination can control COVID-19, dose and type of side effects as explanatory variables.

3. Result : express effect size along with 95% CI for magnitude of outcomes
   Avoid redundancy eg OR, ODDS RATIO??

Response: We have added effect size (Cohen’s d) along with 95% CI

4. Main body: Study design, setting, source population, study pop, inclusion criteria, sample size determination, sampling method, data collection technique, data quality control and data analysis should be stated clearly. identify the type of logistic regression analysis, What are the assumptions of logistic regression model, Have you checked the interactions? indicate the value?

Response: We have rewritten our methods based on above comment. However, we did not check the interactions in logistic regressions.

5. Results: Write your current finding in logical order. Express effect size along with 95% CI for magnitude of outcomes.

Response: We kept our results section as it was. We assume that we have presented results following the sequence of our objectives. We expressed effect size along with 95% CI.

Reviewer #2:

1. In the method section please add at first a small paragraph described the study design and setting, reliability assessment of questionnaire, please provide if do any measure to assess that, provide the cronbach-alfa of the used scale.

Response: We have re-organized method with study design and setting along with other sub-headings suggested by reviewer 1. We validated questionnaire following experts’ opinion and did not conduct any quantitative reliability assessment.

2. sample size : you write that uggested a sample size of 400 at a 95% confidence level with a ±5% precision 210 level [31]. For α = 0.05 and a hypothesized proportion of 0.5, ) but you take a very large size . you can you other assumption to suit your large size,
please provide us paragraph that adjust for this size and mention the sampling technique used.

Response: We have added a sentence to address this comment:

“It was assumed that a sample size of 4175 for this study is adequate to generalize the study findings”.
